# The Road to Recovery: A Pilot Study of Driving Behaviors Following Antibody-Mediated Encephalitis

**DOI:** 10.3389/fneur.2020.00678

**Published:** 2020-07-28

**Authors:** Gregory S. Day, Ganesh M. Babulal, Ganesh Rajasekar, Sarah Stout, Catherine M. Roe

**Affiliations:** ^1^The Charles F. and Joanne Knight Alzheimer Disease Research Center, St. Louis, MO, United States; ^2^Department of Neurology, Washington University School of Medicine, St. Louis, MO, United States

**Keywords:** autoimmune, antibody-mediated, encephalitis, N-methyl-D-aspartate (NMDA) receptor, LGI1, autoantibodies, driving

## Abstract

**Introduction:** Safe driving requires integration of higher-order cognitive and motor functions, which are commonly compromised in patients with antibody-mediated encephalitis (AME) associated with N-methyl-D-aspartate receptors or leucine-rich glioma-inactivated 1 autoantibodies. How these deficits influence the return to safe driving is largely unknown. Recognizing this, we piloted non-invasive remote monitoring technology to longitudinally assess driving behaviors in recovering AME patients.

**Methods:** Five recovering AME patients [median age, 52 years (range 29–67); two females] were recruited from tertiary care clinics at Washington University (St. Louis, MO). Trip data and aggressive actions (e.g., hard braking, sudden acceleration, speeding) were continuously recorded using a commercial Global Positioning System data logger when the patient's vehicle was driven by the designated driver. Longitudinal driving data were compared between AME patients and cognitively normal older adults (2:1 sex-matched) enrolled within parallel studies.

**Results:** Driving behaviors were continuously monitored for a median of 29 months (range, 21–32). AME patients took fewer daily trips during the last vs. the first 6 months of observation, with a greater proportion of trips exceeding 10 miles. Compared to cognitively normal individuals, AME patients were more likely to experience hard braking events as recovery progressed. Despite this, no accidents were self-reported or captured by the data logger.

**Conclusion:** Driving behaviors can be continuously monitored in AME patients using non-invasive means for protracted periods. Longitudinal changes in driving behavior may parallel functional recovery, warranting further study in expanded cohorts of recovering AME patients.

## Introduction

Antibody-mediated encephalitides (AMEs) are a group of increasingly prevalent inflammatory brain disorders that result from antibody-mediated disruption of neuronal cell surface proteins, receptors, or ion channels. AME commonly presents with changes in behavior, psychosis, seizures, memory and cognitive deficits, abnormal movements, dysautonomia, and changes in consciousness (1). Although the potential for meaningful recovery is high in patients who receive early treatment with immunosuppressant agents (1–5), impairments in memory and executive function are well-recognized complications of AME (4, 6–9). How these deficits influence the return to meaningful function in recovering patients is largely unknown, as is the time course and extent of recovery of higher-order function.

Driving is an instrumental activity of daily living that is integral to patient-perceived independence, well-being, autonomy, and quality of life (10, 11). Conversely, forced driving cessation is associated with increased depressive symptomatology, higher rates of admission to institutional care, and social isolation (12, 13). For these reasons, the return to safe driving is a shared priority between patients and their providers. Safe driving requires integration of higher-order cognitive and motor functions, drawing upon heteromodal (association) and unimodal (sensory/motor) cortices and relying heavily on sound judgment and decision-making. Accordingly, changes in driving behavior may provide a real-world correlate for changes in higher-order function associated with neurological diseases (14, 15). Recovering AME patients may experience greater risks following the return to driving due to persistent frontal-executive and memory dysfunction (7, 8). However, little guidance is available to direct clinical recommendations on the return to driving in recovering AME patients.

Recent advances in Global Positioning System (GPS) technology have enabled passive collection of driving data from individuals operating their personal vehicles in their native environments. These advances allow the relationship between longitudinal changes in driving behaviors and higher-order functions to be assessed, with the potential that measures of naturalistic driving behaviors may inform the time course and extent of recovery in AME patients. We leveraged available technology to assess the feasibility of capturing driving behavior over time in a small cohort of patients returning to driving following treatment for AME associated with autoantibodies against N-methyl-D-aspartate receptors (NMDARs) or leucine-rich glioma-inactivated 1 (LGI1) receptors.

## Materials and Methods

### Standard Protocol Approvals, Registrations, and Patient Consents

Patients were enrolled from January to October 2017 within prospective studies permitting longitudinal collection and monitoring of clinical symptoms and signs in recovering AME patients. Written informed consent was obtained from all individuals or their delegates, allowing the collection of relevant clinical information and installation and remote monitoring of driving behaviors using a commercial-off-the-shelf GPS data logger together with naturalistic driving methodology (15, 16)—termed the Driving Real World In-Vehicle Evaluation System [DRIVES (17)]. The Washington University School of Medicine Institutional Review Board approved all study procedures.

### Participant Selection, Evaluation, and Follow-Up

Patients with AME were admitted to study hospitals and thoroughly evaluated by experienced clinicians. All patients met the criteria for definite AME, including presentation with the subacute-onset of memory deficits, mental status change, or psychiatric symptoms, with at least one of new focal central nervous system findings, unexplained seizures, cerebrospinal fluid (CSF) pleocytosis, or MRI features suggestive of encephalitis. Immunoglobulin G (IgG) autoantibodies were identified against central nervous system NMDAR in the CSF or LGI1 receptors in the CSF or serum, and alternate causes of impairment were excluded (18). Antibody testing was performed at the Mayo Clinic Neuroimmunology Laboratory (Rochester, Minnesota) using indirect immunofluorescence (specimen applied to frozen mouse composite tissue, washed and treated with fluorescein-conjugated IgG), cell binding, Western blot, and radioimmune assays. Investigations and immunomodulatory treatments were prescribed by treating physicians in accordance with clinical indications. Outpatient follow-up was provided *via* the Washington University Rapidly Progressive Dementia Clinic. Cognitive functions were prospectively assessed at clinical visits with the global Clinical Dementia Rating (CDR)—a widely used, validated, and reproducible measure of cognitive function (19)—and standardized bedside measures of global cognitive function, including the Montreal Cognitive Assessment [MoCA (20)] and Mini-Mental State Examination [MMSE (21)]. Disability was assessed using the modified Rankin scale [mRS (22)]—a common outcome measure in AME (2, 3, 5). All participants completed the Trails A and B tasks, recognizing that performance on these measures may predict performance on on-road driving assessments (23).

Community-dwelling cognitively normal (CN) participants (*n* = 10; 2:1 sex-matched; median age = 68 years, range 65–70) were recruited from longitudinal studies of memory and aging at the Washington University Knight Alzheimer Disease Research Center (St. Louis, MO). Clinical and neuropsychological assessments were completed as previously described (15, 24). All participants were CN [global CDR = 0 (19)], had a valid driver's license, and reported driving at least once per week.

### Measurement of Naturalistic Driving Behaviors

All patients reported ≥5 years of pre-illness on-road driving experience, maintained an active driver's license, and were cleared to return to driving by their treating neurologist, referencing prevailing laws. No patient had electrographically confirmed seizures in the hospital. Prior to the return to driving, a commercial-off-the-shelf GPS data logger (DRIVES; G2 Tracking Device™, Azuga Inc., San Jose, CA) was plugged into the participant's vehicle's onboard diagnostics-II (OBD-II) port. Within 1 min of installation, the chip accessed available satellites for orientation and synchronization and began transmitting data to servers in San Jose (CA) *via* Bluetooth Low Energy using available cell phone towers. Data were aggregated daily by the vendor and made available for download *via* secured servers. Study participants (AME patients and CN individuals) agreed to carry a credit card-sized Bluetooth Low Energy device (Azuga Inc., San Jose, CA) in their wallet or purse. This device automatically paired with the chip when the participant was in the driver's seat, identifying the participant as the driver.

Using the DRIVES methodology, data were collected every 30 s while the vehicle was driven by the study participant, recording date and time, latitude, longitude, odometer reading, speed, and event type (16). Vehicle speed was compared to the posted speed limit in a given region. “Speeding” events were encoded whenever vehicle speed exceeded the posted limit by 6 miles per hour. Rapid changes in vehicle speed were recorded as hard braking or sudden acceleration events (16). These “aggressive driving” events (speeding, hard braking, sudden acceleration) were recorded anytime they occurred during a trip, regardless of the data sampling interval. Trip data were also captured, including trip date and start time, starting and ending locations, the duration in seconds and length of the trip, the mean and maximum vehicle speeds, and the number of aggressive driving events. A “trip” was defined from the period of “ignition on” to “ignition off” (e.g., a return excursion to the grocery store without any other stops would be considered two trips). Data logger data were aggregated by month, so that an individual followed for 1 year would have 12 data points across time for each variable.

### Statistical Analyses

Data were collected and managed securely using a research electronic data capture tool [REDCap (25)]. Individual performance on standardized cognitive tests were compared with expected performance derived from age- and education-matched normative data for the MoCA (20), MMSE (26), and Trails A/B tasks (23). Briefly, *Z*-scores were calculated on a test-by-test basis by subtracting the reported population mean from the patient's raw score and dividing the difference by the population standard deviation. *P*-values were inferred from the standard normal distribution, with *p* < 0.05 deemed significant. Driving behaviors in AME patients were dichotomized, comparing between “early” (first 6 months following the return to driving) and “late” (last 6 months of observation) periods using the S-statistic. Differences in longitudinal driving behaviors between AME patients and CN individuals were quantified using univariate linear mixed models, querying the interaction between cohort and the week number. A more permissive *p*-value was selected (*p* < 0.1) for population comparisons, given the exploratory nature of these analyses in a small cohort of AME patients. Statistical analyses were conducted in SAS version 9.4 (SAS Institute, Cary, NC), while data analysis and management for spatial operations used ArcGIS 10.3.1 and the ArcPy Python site package (Environmental Systems Research Institute, Redlands, CA).

## Results

Driving behaviors were assessed in five recovering AME patients [median age, 52 years (range 29–67); two females]. Demographic, clinical characteristics, and results of investigations and treatments are detailed in Table 1. All patients met diagnostic criteria for definite AME (18), with presentations and clinical courses consistent with AME associated with NMDAR (18) or LGI1 (3) autoantibodies. Maximal illness severity ranged from mild (mRS ≤ 2; e.g., Case C) to severe (mRS = 5; Case A). No patient had a disease-associated tumor. First-line immunotherapies, including high-dose methylprednisolone [1 g intravenously (IV) over 5 days] or intravenous immunoglobulin (2 g/kg over 5 days), were provided to all patients within 1 week of hospital admission. Two patients with persistent impairment were additionally treated with rituximab (375 mg/m^2^ IV weekly × 4). Patients with AME associated with autoantibodies against LGI1 antigens were continued on oral steroids (1 mg/kg daily) upon hospital discharge, with the dosage tapered over 3 months.

**Table 1:** Demographic features, clinical presentation, investigations, and treatments in AME patients.

	**Clinical features**		**Investigations**			**Time to treatment, weeks**		**Illness severity**		
**Case/age,^†^ sex**	**Presenting symptoms**	**Exam findings**	**CSF, nucleated cells/mm^**3**^**	**Brain MRI**	**Antibody**	**Steroids/IVIg**	**Rituximab**	**Admission duration, weeks**	**mRS (nadir)**	**gCDR (nadir)**
A/29F	Psychoses, memory loss, AMS	No focal deficits	21	Normal	NMDAR (CSF)	2	2.9	9	5	3
B/52F	Psychoses, memory loss, AMS	Expressive aphasia, right hand clumsiness and apraxia	35	Left cortical T2-FLAIR hyperintensity and diffusion restriction	NMDAR (CSF)	30	NA	3	3	3
C/37M	Memory loss, painful paresthesias	No focal deficits	9	Normal	LGI1 (serum)	52	66.1	1	2	0.5
D/63M	Visual hallucinations, headache, fever	Distal > proximal weakness, areflexia	62	Normal	LGI1 (serum)	4	NA	2	4	0.5
E/67M	Agitation, memory loss, AMS, symptomatic bradycardia	No focal deficits, hyponatremia (unexplained)	5	Normal	LGI1 (serum)	40	NA	1	2	2

*AME, antibody-mediated encephalitis; AMS, altered mental status; CSF, cerebrospinal fluid; FLAIR, fluid-attenuated inversion recovery; gCDR, global Clinical Dementia Rating (19); IVIg, intravenous immunoglobulin (0.4 g/kg ×5 days); LGI1, leucine-rich glioma-inactivated 1; MRI, magnetic resonance imaging; mRS, modified Rankin scale (22); NMDAR, N-methyl-D-aspartate receptor; PLEX, plasmapheresis/plasma exchange (10 treatments); rituximab, 375 mg/m^2^ IV ×4 weeks; steroids, intravenous methylprednisolone (1 g per day ×5 days)*.

† *Age at DRIVES install*.

Recovering AME patients returned to driving a median of 5.7 (range, 1–16) weeks following admission to the hospital (34 weeks from symptom onset, range 12–68), by which time all patients had “good” motor outcomes, defined as mRS ≤ 2. Cognitive function and processing speed were further assessed using the global CDR and bedside measures (Table 2). Patients with AME associated with LGI1 autoantibodies had very mild impairment in cognitive function (CDR = 0.5); patients with NMDAR autoantibodies were CN (CDR = 0). Performance on the MoCA was substantially lower than expected for age and education in 3/5 (60%) of AME patients (*Z* ≤ −1.96; *p* < 0.05). Only Case D exhibited impairment on the MMSE (*Z* = −2.3; *p* = 0.02). All patients completed the Trails A task as expected. Cases C (*Z* = −3.6; *p* < 0.001) and E (*Z* = −5.6; *p* < 0.001) were substantially slower in completing Trails B.

**Table 2:** Cognitive and motor outcome measures at the time of the return to driving in AME patients.

**Case**	**Weeks from admission**		**mRS**		**gCDR**		**MoCA, score (*****Z*****)**		**MMSE, score (*****Z*****)**		**Trails A, seconds (*****Z*****)**		**Trails B, seconds (*****Z*****)**	
	**T1**	**T2**					**T1**	**T2**	**T1**	**T2**	**T1**	**T2**	**T1**	**T2**
A	12.1	15.1	2	0	0	0	30 (1.2)	30 (1.2)	30 (1.1)	30 (1.1)	29 (−0.9)	30 (−1.0)	55 (−0.5)	42 (0.6)
B	4.4	20.4	1	0	0	0	**23** **(−2.0)**	NA	28 (0)	30 (0.9)	34 (−0.2)	32 (0)	59 (0.3)	58 (0.4)
C	15.9	33.6	1	1	0.5	0.5	**17** **(−4.7)**	NA	25 (−1.7)	27 (−0.6)	37 (−0.8)	32 (−0.3)	**118** **(−3.6)**	**101** **(−2.6)**
D	5.7	74.7	2	1	0.5	0	**22** **(−2.5)**	NA	**26** **(−2.3)**	NA	31 (0.2)	NA	86 (−0.6)	NA
E	0.6	17.0	2	1	0.5	0	25 (−1.1)	NA	28 (−1.0)	30 (1.0)	28 (0.9)	36 (−0.3)	**119** **(−5.6)**	**103** **(−3.9)**

*Z-scores for neuropsychological testing were calculated by comparing raw scores to age- and education-matched norms. Z-scores were calculated on a test-by-test basis by subtracting the reported population mean from the patient's raw score and dividing the difference by the population standard deviation. Negative Z-scores reflect worse-than-expected patient performance; positive Z-scores reflect better-than-expected patient performance. P-values were inferred from the standard normal distribution. P values < 0.05 are bolded*.

*Trail Making Tests A and B are scored in seconds [higher values indicate worse performance (23)]*.

*AME, antibody-mediated encephalitis; gCDR, global Clinical Dementia Rating (19); MMSE, Mini-Mental State Examination (21, 26); MoCA, Montreal Cognitive Assessment (20); mRS, modified Rankin scale (22); NA, not available (test not performed)*.

Driving behaviors were monitored for a median of 29 months (range, 21–32). No accidents were reported by study participants or captured by the DRIVES. Compared to the initial 6 months following the return to driving, AME patients took fewer daily trips in the last 6 months of observation, with an increased tendency to take longer trips (≥10 miles; Table 3). No differences were observed in the relative frequency of behaviors associated with aggressive driving in the early and late observation periods. When longitudinal driving behaviors were compared between AME patients and a 2:1 sex-matched cohort of older CN individuals, recovering AME patients experienced more hard braking events per trip with weeks from recovery (slope = 0.18 ± 0.07), while CN individuals experienced fewer events (slope = −0.003 ± 0.04; *p* = 0.08; Table 4). No differences were observed in other driving behaviors between AME patients and CN individuals.

**Table 3:** Early and late driving behaviors in AME patients.

	**Measure**	**First 6 months, mean (SD)**	**Last 6 months, mean (SD)**	**S-statistic**	***p*-value**
Travel patterns (per month)	Total miles driven, *n*	6,933.9 (4,841.7)	5,768.3 (1,666.2)	0.5	>0.99
	Miles/trip	11.0 (7.1)	11.8 (6.1)	−0.5	>0.99
	**Trips** **≥10 miles, %**	**23.1** **(13.5)**	**31.2** **(21.2)**	**−7.5**	**0.06**
	Total Trips, *n*	663.2 (317.6)	600.6 (293.6)	3.5	0.44
	**Daily trips**, ***n***	**5.0** **(1.2)**	**4.6** **(1.0)**	**7.5**	**0.06**
	Trips in daylight, *n*	80.6 (10.7)	78.5 (12.5)	2.5	0.63
	Trips at night, *n*	7.38 (4.7)	6.7 (4.4)	0.5	>0.99
Aggressive behaviors (per month)	Trips with any events, *n*	22.0 (19.3)	30.9 (27.6)	−4.5	0.31
	Hard braking, *n*	7.01 (6.7)	12.8 (16.9)	−2.5	0.63
	Speeding, *n*	17.99 (17.3)	25.2 (22.2)	−4.5	0.31
	Sudden acceleration, *n*	2.88 (4.4)	3.4 (6.6)	2.5	0.65
	Hard braking, events per trip	8.414 (8.9)	19.2 (27.5)	−2.5	0.63
	Speeding, events per trip	118.2 (85.1)	120.9 (110.2)	−0.5	>0.99
	Sudden acceleration, events per trip	3.16 (4.76)	4.12 (7.45)	2.50	0.63

*AME, antibody-mediated encephalitis; SD, standard deviation*.

*Comparisons where p < 0.1 are bolded*.

**Table 4:** Slopes of linear mixed models describing the interaction between driving behaviors and time from the return to driving (weeks) in AME patients and CN individuals.

	**Measure**	**AME Patients, slope (SE)**	**CN Individuals, slope (SE)**	***p*-value**
Travel patterns	Miles driven	−0.004 (0.73)	−0.20 (0.42)	0.78
	Miles/trip	0.01 (0.03)	−0.01 (0.02)	0.81
	Proportion of trips ≥10 miles	0.09 (0.06)	−0.05 (0.03)	0.12
	Number of trips	−0.004 (0.04)	−0.04 (0.02)	0.19
	Daily trips	−0.005 (0.004)	−0.004 (0.002)	0.13
	Trips in daylight	−0.02 (0.05)	0.04 (0.03)	0.45
	Trips at night	0.01 (0.02)	0.01 (0.01)	0.45
Aggressive behaviors	Number of trips with any events	0.13 (0.08)	−0.05 (0.04)	0.14
	Hard braking	0.08 (0.04)	−0.05 (0.04)	0.14
	Speeding	0.11 (0.08)	−0.04 (0.04)	0.21
	Sudden acceleration	0.02 (0.02)	−0.01 (0.01)	0.46
	**Hard braking, events per trip**	**0.18** **(0.07)**	**−0.003** **(0.04)**	**0.08**
	Speeding, events per trip	0.29 (0.37)	0.02 (0.21)	0.73
	Sudden acceleration, events per trip	0.04 (0.03)	−0.02 (0.02)	0.37

*AME, antibody-mediated encephalitis; CN, cognitively normal; SE, standard error*.

*Comparisons where p < 0.1 are bolded*.

Patients were reassessed a median of 16.4 weeks (range, 3–69) following the return to driving [median 57.4 weeks (range, 17–86) from symptom onset], by which time all patients had successfully returned to prior vocational or educational function and had tapered off immunotherapies. The global CDR was 0 (CN) in all patients, except for Case C who complained of isolated short-term memory deficits (CDR 0.5, memory only). The mRS ranged from 0 to 1 (median = 1). Cognitive testing was completed in four of five patients (omitted in Case D), with improvement noted in all domains in all patients. Performance on the Trails B test remained slower than expected in two, Cases C (*Z* = −2.6, *p* < 0.01) and E (*Z* = −3.9, *p* < 0.001); otherwise, performance was within the range expected for age and education.

## Discussion

Frontal-executive and memory dysfunction are well-recognized complications of AME associated with NMDAR and LGI1 autoantibodies, with deficits attributed to changes in the density of cell surface receptors (27–29), brain structure (7, 30), and network integrity (7, 8, 31–33). Although these changes may be reversible, the time course and extent over which recovery occurs are largely unknown. Safe driving requires continuous integration of complex cognitive and motor functions that are widely distributed throughout the brain (34, 35). It is not surprising, therefore, that dynamic changes in driving behaviors were observed in the small cohort of AME patients included in this pilot study.

The tendency to take more frequent, shorter trips upon the return to driving may reflect self-regulation by AME patients—referring to the process whereby drivers intentionally avoid driving in situations considered to be challenging [e.g., long travel, driving within congested urban areas or on freeways (36)]. When aptly deployed, these adaptive strategies may extend the duration over which individuals may drive safely and maintain independence (37, 38). Detection of these changes may identify individuals at-risk for brain pathology that may compromise driving ability. In a longitudinal study measuring objective driving behaviors in older CN community-dwelling adults, drivers with early (i.e., “preclinical”) brain changes related to Alzheimer disease exhibited decreased driving space and exposure and had a lower number of trips with “aggressive behaviors” (especially hard braking and sudden acceleration) than their Alzheimer disease-free counterparts (15). In contrast to patients with neurodegenerative diseases, the cognitive and motor deficits associated with AME may resolve with time from treatment. Consistent with this, recovering AME patients were more likely to take longer trips with more frequent hard braking episodes later in the recovery period. These changes could suggest increasing risky or aggressive behaviors. Alternatively, the emergence of hard braking episodes in recovering patients taking longer trips may reflect progressive normalization of driving behaviors in patients returning to professional and personal routines. This may include travel within urban centers and other areas (e.g., shopping centers, parking lots) where hard braking may be required to prevent accidents while navigating amid higher-density traffic.

Although preliminary, these results suggest that DRIVES may be used to measure functional recovery—not just decline. Access to an objective and dynamic measure of driving safety would convey substantial advantages over existing time-consuming and costly approaches used to assess driving safety, including direct observation of driving behaviors in structured or simulated environments (14, 35, 39, 40). If validated in larger numbers of patients, DRIVES may permit continuous, non-invasive, and objective measurement of complex cognitive and motor behaviors across protracted periods of time. In turn, these findings may inform the timeline for the safe return to driving in AME patients; facilitate evaluation of modifiable patient-, disease-, and treatment-specific factors that associate with recovery; and inform the association between driving behaviors and neurologic sequelae in recovering AME patients.

The interpretation of study findings is limited by the small number of patients enrolled in this pilot study conducted at a single academic medical center. Findings need to be validated in larger numbers of patients with AME associated with autoantibodies against NMDAR, LGI1, and other antigens, acknowledging the broad variability in the clinical phenotype and long-term outcomes in AME (1–6, 9) and the low likelihood of detecting rare events (i.e., motor vehicle accidents) in small cohorts of patients. Future studies would also benefit from recruitment of age-matched comparison cohorts. As older age is associated with increasing age performance inconsistencies (41), the use of a convenience cohort comprised of older CN individuals may have decreased the ability to detect longitudinal differences between AME patients and CN individuals in this study. Non-invasive monitoring was performed across prolonged periods of time to minimize the contributions of observation bias to the results of this pilot study. However, it is possible that AME patients modified driving behaviors in response to monitoring. The effect of monitoring could be evaluated through future studies that vary the degree of invasive monitoring. Integration of additional tools that permit recording of the driver's field of view would also allow direct evaluation of “near misses” and other risky behaviors in recovering patients, as well as other environmental factors that may influence driving performance (e.g., presence or absence of passengers, use of radio). Such tools may assist with determining whether trends in behavior (e.g., hard braking events) represent defensive maneuvers executed by drivers commuting to work in congested urban areas (a correlate for functional improvement or normalization of driving behaviors) or unsafe behaviors observed in distracted drivers (a correlate for persistent dysfunction).

Limitations notwithstanding, the DRIVES provided robust information on driving behaviors in a small cohort of recovering AME patients. These results suggest that driving behaviors in AME patients can be continuously monitored using non-invasive means across protracted periods of time. Longitudinal changes in driving behavior may parallel functional recovery in AME, warranting further study in expanded cohorts of patients who have been approved to return to driving by their treating physicians, referencing prevailing laws and recommendations.

## Data Availability Statement

Anonymized data will be shared with qualified investigators upon request to the corresponding author.

## Ethics Statement

The studies involving human participants were reviewed and approved by the Washington University School of Medicine Institutional Review Board. The patients/participants provided their written informed consent to participate in this study. Written informed consent was obtained from the individual(s) for the publication of any potentially identifiable images or data included in this article.

## Author Contributions

GD participated in study design, provision of patient care, acquisition and interpretation of clinical data, statistical analysis, and drafting, revision, and finalization of the manuscript. GB participated in acquisition and interpretation of driving data, statistical analysis, and drafting, revision, and finalization of the manuscript. GR participated in statistical analysis and revision and finalization of the manuscript. SS participated in acquisition of driving data and revision and finalization of the manuscript. CR participated in acquisition and interpretation of driving data, statistical analysis, and revision and finalization of the manuscript. All authors contributed to the article and approved the submitted version.

## Conflict of Interest

GD is supported by the National Institutes of Health/National Institute on Aging (K23AG064029). He serves as a topic editor on dementia for DynaMed Plus (EBSCO Industries, Inc), is the clinical director for the Anti-NMDA Receptor Encephalitis Foundation (uncompensated), has provided record review and expert medical testimony on legal cases pertaining to management of Wernicke encephalopathy, and holds stocks (>$10,000) in ANI Pharmaceuticals (a generic pharmaceutical company). The remaining authors declare that the research was conducted in the absence of any commercial or financial relationships that could be construed as a potential conflict of interest.
